# Spousal Concordance in Depressive Symptoms and the following Cognitive Impairment and Mortality

**DOI:** 10.1093/geroni/igaf122.2910

**Published:** 2025-12-31

**Authors:** Xuechun Wang

**Affiliations:** Peking University, Beijing, Beijing, China (People’s Republic)

## Abstract

**Objective:**

Prior studies have focused on individual depression’s health impact, with less attention to spousal depressive symptoms and their health effects. This study examines the association between spousal depressive symptom concordance, mild cognitive impairment (MCI) and mortality risk among middle-aged and older couples.

**Method:**

We analyzed five waves of China Health and Retirement Longitudinal Study (2011 to 2020) data. A group-based multi-trajectory model identified trajectories of spousal depressive symptoms. Cox proportional hazards models examined associations between depressive concordance, cognitive decline, and mortality risk. A multi-state survival model estimated transition risks from normal cognition to MCI and to mortality. Linear mixed-effects models evaluated depressive concordance and cognitive functions (mental intactness and episodic memory).

**Results:**

Among 11912 respondents, four depressive symptom trajectories were identified: Stable Low-Low (50.29%), Stable High-Mild (21.25%), Stable Mild-High (20.92%) and Stable High-High (7.54%). Compared with baseline neither-depressive-symptoms group, couples in the both-depressive-symptoms group exhibited the highest MCI (HR = 1.33,95% CI: [1.22, 1.45]) and mortality (HR = 1.36, 95% CI: [1.15, 1.62]) risk, with greater transition risks to MCI (β = 0.31, 95% CI: [0.09, 0.53]) and to mortality (β = 0.19, 95% CI: [0.04, 1.35]). They also exhibited poorer episodic memory (β = -0.30, 95% CI: [-0.37, -0.24]) and mental intactness (β = -0.60, 95% CI: [-0.69, -0.51]), with faster decline in episodic memory (β=-0.01, 95% CI: [-0.03, -0.0002]).

**Conclusion:**

Spousal depressive symptom concordance remained stable over time. Couples with high-symptom patterns had increased risks of MCI and mortality, highlighting the need for couple-centered mental health interventions.

